# Expression and Purification of C-Peptide Containing Insulin Using* Pichia pastoris* Expression System

**DOI:** 10.1155/2016/3423685

**Published:** 2016-08-04

**Authors:** Mohammed N. Baeshen, Thamer A. F. Bouback, Mubarak A. Alzubaidi, Roop S. Bora, Mohammed A. T. Alotaibi, Omar T. O. Alabbas, Sultan M. Alshahrani, Ahmed A. M. Aljohani, Rayan A. A. Munshi, Ahmed Al-Hejin, Mohamed M. M. Ahmed, Elrashdy M. Redwan, Hassan A. I. Ramadan, Kulvinder S. Saini, Nabih A. Baeshen

**Affiliations:** ^1^Department of Biological Sciences, Faculty of Science, King Abdulaziz University, P.O. Box 80203, Jeddah 21589, Saudi Arabia; ^2^Department of Biological Sciences, Faculty of Science, University of Jeddah, P.O. Box 80327, Jeddah 21589, Saudi Arabia; ^3^Department of Biotechnology, Eternal University, Baru Sahib, Himachal Pradesh 173 101, India; ^4^Nucleic Acids Research Department, Genetic Engineering and Biotechnology Research Institute (GEBRI), City of Scientific Research and Technology Applications, Alexandria 21934, Egypt; ^5^Protein Research Department, Genetic Engineering and Biotechnology Research Institute, City of Scientific Research and Technology Applications, Alexandria 21934, Egypt; ^6^Cell Biology Department, Genetic Engineering and Biotechnology Division, National Research Centre, Tahrir Street, Dokki, Cairo 12311, Egypt

## Abstract

Increase in the incidence of Insulin Dependent Diabetes Mellitus (IDDM) among people from developed and developing countries has created a large global market for insulin. Moreover, exploration of new methods for insulin delivery including oral or inhalation route which require very high doses would further increase the demand of cost-effective recombinant insulin. Various bacterial and yeast strains have been optimized to overproduce important biopharmaceuticals. One of the approaches we have taken is the production of recombinant human insulin along with C-peptide in yeast* Pichia pastoris*. We procured a cDNA clone of insulin from Origene Inc., USA. Insulin cDNA was PCR amplified and cloned into yeast vector pPICZ-*α*. Cloned insulin cDNA was confirmed by restriction analysis and DNA sequencing. pPICZ-*α*-insulin clone was transformed into* Pichia pastoris SuperMan*_*5*_ strain. Several Zeocin resistant clones were obtained and integration of insulin cDNA in* Pichia* genome was confirmed by PCR using insulin specific primers. Expression of insulin in* Pichia* clones was confirmed by ELISA, SDS-PAGE, and Western blot analysis. In vivo efficacy studies in streptozotocin induced diabetic mice confirmed the activity of recombinant insulin. In conclusion, a biologically active human proinsulin along with C-peptide was expressed at high level using* Pichia pastoris* expression system.

## 1. Introduction

Diabetes mellitus is a metabolic disorder which is characterized by hyperglycemia due to low level or complete deficiency of insulin hormone. Type 1 diabetes is triggered by insulin deficiency, whereas, in most cases, type 2 diabetes is caused by insulin resistance along with insufficient insulin secretion. A chronic hyperglycemic condition is more often associated with vascular complications, particularly involving the eyes, kidneys, hearts, blood vessels, and nerves [1]. Presently, diabetes is one of the leading causes of death globally [2].

Insulin is a hormone which is synthesized by the *β* cells of the pancreatic islets of Langerhans. It plays a key role in modulating blood glucose levels. It is produced as a 108-amino acid long single polypeptide precursor, preproinsulin, containing a signal sequence at its N-terminus which is cleaved by a specific endopeptidase. Proinsulin is further proteolytically processed in the coated secretory granules, producing a mature insulin and 34 amino acids of C-peptide. Mature insulin has molecular weight of 5807 daltons and is comprised of two polypeptides connected by two interchain disulphide bonds. The A chain is composed of 21 amino acids, whereas the B chain contains 30 amino acids [3, 4].

The initial approach for commercial production of recombinant human insulin, employed by scientists in Genentech, required transformation of the cDNA encoding for the human insulin A and B chains separately into* E. coli* cells (K12). These cells were then cultured individually in large fermenters and both insulin peptides were purified using chromatographic methods. The A and B chains of insulin were then incubated together under oxidizing conditions to promote folding and interchain disulphide bond formation, hence forming an active human insulin [5, 6]. An alternative approach, adopted by Eli Lilly Research Laboratories, involved expression of a single polypeptide of human proinsulin in* E. coli*, followed by purification and removal of C-peptide by proteolytic cleavage to yield the active insulin [7, 8]. Currently,* E. coli* and* Saccharomyces cerevisiae* are the preferred expression host for commercial production of recombinant human insulin for the therapeutic use in humans [9–11].

The main objective of our work is to explore other alternate hosts such as* Pichia pastoris* for large scale production of recombinant human insulin.* Pichia pastoris*, like other eukaryotes, offer several advantages including proper protein folding, protein processing, and posttranslational modifications. Moreover, like* E. coli* and* Saccharomyces cerevisiae*, it is very easy to manipulate genetically and very cost-effective expression system for bulk production of recombinant proteins.* Pichia pastoris* is a methylotrophic yeast which metabolizes methanol as a carbon source. Methanol is oxidized to formaldehyde by the activity of the enzyme alcohol oxidase (*AOX1*) in presence of oxygen. Since alcohol oxidase has poor affinity for oxygen, cells compensate it by producing large amount of the enzyme. Expression of alcohol oxidase is induced by methanol to very high levels, approximately, more than 30% of total protein in cytosol of* Pichia* cells. The promoter controlling the synthesis of alcohol oxidase is used for expression of recombinant proteins in* Pichia pastoris* [12, 13]. Besides high expression levels,* Pichia pastoris* has another significant advantage in glycosylation pattern of heterologous protein, in comparison to* Saccharomyces cerevisiae*. It has been shown that heterologous proteins in* Pichia* are not hyperglycosylated as length of the oligosaccharide chains added to proteins is around 8–14 mannose residues per side chain in* Pichia* which is very short as compared to that in* S. cerevisiae*, that is, approximately ~50 to 150 mannose residues per side chain [14, 15]. Moreover, unlike in* Pichia*, glycosylated proteins produced from* S. cerevisiae* have terminal *α*1,3 glycan linkages which are responsible for the hyperantigenic property of these proteins and thus rendering them unsuitable for therapeutic applications in human [16]. Hence, recombinant glycoprotein produced in* Pichia* may be more similar to the glycoprotein structure of higher eukaryotes. These important features make* Pichia* an attractive expression host for large scale production of recombinant proteins for therapeutic use.

## 2. Materials and Methods

### 2.1. Cloning of Insulin cDNA in* Pichia* Vector

Plasmid pCMV6-XL harboring proinsulin cDNA was procured from Origene Inc., USA, and used as a template to amplify insulin cDNA. For secreted expression of insulin, there are forward primer 5′-tgatgaattctttgtgaaccaacacctgtgcgg-3′ and reverse primer 5′-acttctcgagagttgcagtagttctccagctggta-3′. Proinsulin cDNA was PCR amplified using insulin specific primers and High Fidelity pfu. PCR amplified proinsulin cDNA was double-digested with* Eco*RI and* Xho*I and ligated with the pPICZ-alpha vector double-digested with* Eco*RI and* Xho*I. Ligated mixture was transformed into competent* E. coli* cells. Several Zeocin resistant transformants were obtained on LB-Zeocin plate. Few clones were screened for the presence of proinsulin cDNA in pPICZ-alpha vector by restriction mapping. Proinsulin cDNA cloned in* Pichia* vector pPICZ-*α* was further confirmed by DNA sequencing.

### 2.2. Transformation of* Pichia pastoris*

pPICZ-*α*-proinsulin construct was transformed into* Pichia pastoris SuperMan*_*5*_ strain by electroporation. For efficient integration of recombinant construct into* Pichia* genome, pPICZ-*α*-insulin plasmid DNA was linearized with SacI. Pichia strain was transformed with the insulin construct by electroporation as instructed by manufacturer. Briefly, 0.5 mL of overnight grown culture of* Pichia pastoris SuperMan*_*5*_ strain was inoculated into 500 mL of YPD media in a 2-liter flask and allowed to grow at 30°C to an OD_600_ ~ 1.3–1.5. Cells were centrifuged at 1500 ×g at 4°C for 5 min and cell pellet was washed with 250 mL of sterile ice-cold water twice and once with 20 mL of ice-cold 1 M sorbitol. Cells were centrifuged at 1500 ×g at 4°C for 5 min and cell pellet was resuspended in 1 mL of ice-cold 1 M sorbitol and kept on ice for 10 min. 100 *μ*L of cells was mixed with 10 *μ*g of linearized pPICZ-insulin plasmid DNA and transferred to an ice-cold 0.2 cm electroporation cuvette and kept on ice for 5 min. Cells were electroporated at 540 V, 25 *μ*F, resistance set to infinite (∞), and pulse time of 15–20 ms. Immediately, 1 mL of ice-cold 1 M sorbitol was added to the cuvette and cells were transferred to a sterile 15 mL tube and incubated at 30°C without shaking for 2 hrs. 50–100 *μ*L cell suspension was plated on YPDS plates containing 100 *μ*g/mL Zeocin incubated at 30°C for 3–5 days until colonies form. For selecting putative multicopy recombinants, transformation mix was plated on increasing concentration of Zeocin, that is, 500–2000 *μ*g/mL.

### 2.3. Expression and Purification of Insulin Protein

Using a single colony, 25 mL of MGYH media (minimal medium containing glycerol and histidine) was inoculated in a 250 mL baffled flask and grown at 28–30°C in a shaking incubator (250–300 rpm) until culture reaches an OD_600_ = 2–6 (approximately 16–18 hours). The cells were centrifuged at 1,500–3,000 ×g for 5 min at room temperature. Supernatant was discarded and cell pellet was resuspended to an OD_600_ of 1.0 in MMH (minimal medium containing methanol) medium to induce expression (approximately 100–200 mL). Culture was placed in a 1-liter baffled flask and kept in incubator. 100% methanol was added to a final concentration of 0.5% methanol after every 24 hours for induction. At the various time interval, 1 mL of the culture was transferred to a 1.5 mL microcentrifuge tube. These samples were used to analyze expression levels of proinsulin and also determine the optimal time postinduction to harvest the cells. Cells were harvested in a tabletop microcentrifuge for 2-3 minutes at room temperature. For secreted expression, the supernatant was transferred to a separate tube. The supernatant and the cell pellets were stored at −80°C until further use. Insulin protein concentration in the culture supernatant before and after induction was estimated by using micro protocol of Pierce Coomassie (Bradford) Protein Assay Kit according to Thermo Scientific instruction. The cell pellets and supernatants were analyzed for protein expression by SDS-PAGE, Western blot, and ELISA.

### 2.4. ELISA

The in-house expressed insulin and commercial insulin were used for coating the Costar ELISA plates in carbonate/bicarbonate buffer (pH 9.6) for 1 hr at 37°C and followed by incubation at 4°C for overnight. The plates were blocked with 1% BSA + 0.1% tween 20. Proteins were detected by using specific monoclonal antibody against recombinant human insulin (Takara, Otsu, Shiga, Japan) at dilution 1 : 1000 in washing buffer for 1 hr and then reacted with goat anti-mouse-alkaline phosphatase conjugated antibody for 1 hr at dilution 1 : 2000 in washing buffer. 50 *μ*L/well of* p*-nitrophenyl phosphate (*p*NPP) substrate was added to each welland reading was taken at 495 nm [11].

### 2.5. *In Vivo* Efficacy Studies in Streptozotocin Induced Diabetic Mice

 50 seven-week-old Swiss outbred mice having weight of 18–20 grams/mice were used in this study. Mice were divided into 5 groups, each group comprising of 10 mice (5 females and 5 males in separate cages) as follows: (1) Control Group I (no treatment); (2) Control Group II (streptozotocin + commercial insulin, NovoRapid® FlexPen®); (3) Control Group III (streptozotocin, no insulin treatment); (4) Experimental Group IV (streptozotocin + proinsulin, produced in the lab by yeast after 144 hours); (5) Experimental Group V (no streptozotocin, proinsulin produced in the lab by yeast after 144 hours). All the animals were kept in their cages for 5–7 days before streptozotocin injection. The glucose level was estimated in all animals before injection. Diabetes was induced by using low dose of streptozotocin in mice (1.75 mg/mice) for Groups II, III, and IV and all the animals were kept for 10–12 days before treatment with the insulin products. The glucose level was estimated in all animals before treatment with insulin products. Antidiabetic agents, that is, commercial insulin for Group II and human proinsulin for Groups IV and V, were injected into respective animals (injection by 0.6 U/100 g). The glucose level was estimated in all five groups after 2, 4, and 6 hrs.

## 3. Results and Discussion

### 3.1. Cloning of Insulin cDNA in Pichia Vector pPICZ-*α*

**Figure 1:**
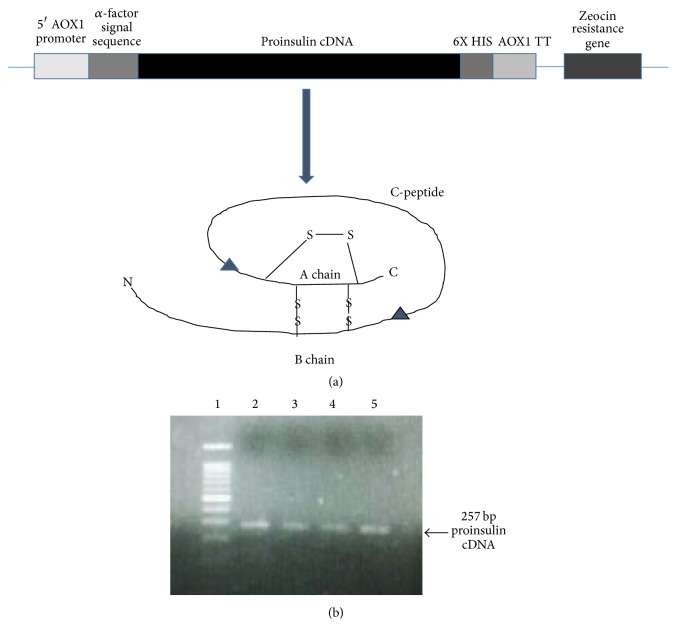
(a) Schematic diagram showing construction of recombinant pPICZ-*α*-proinsulin plasmid. (b) Agarose gel electrophoresis showing PCR amplification of proinsulin cDNA. Lane 1: DNA marker; lanes 2–5: PCR amplified proinsulin cDNA.

**Figure 2:**
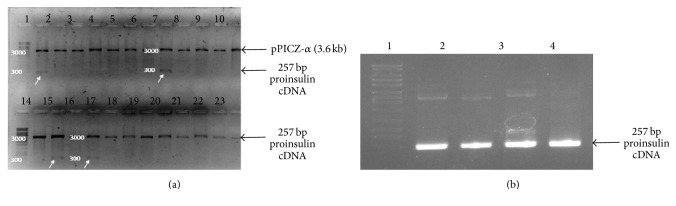
(a) Agarose gel electrophoresis for screening of positive* E. coli* clones harboring recombinant pPICZ-*α*-proinsulin. Lanes 1 and 14: DNA marker; lanes 2 to 13 and 15 to 26: plasmid DNA isolated from Zeocin resistant* E. coli* transformants and double-digested with* Eco*RI and* Xho*I. (b) Agarose gel electrophoresis for confirmation of clones by PCR. Lane 1: DNA marker; lanes 2–5: positive clones 1 to 4.

Plasmid pCMV6-XL harboring proinsulin cDNA was procured from Origene Inc., USA. Insert DNA (approx. 500 bp) was confirmed by restriction digestion with* Not* I as there are two sites for* Not* I flanking the insert DNA. This clone was used as a template to amplify the proinsulin cDNA. A 257 bp proinsulin cDNA was PCR amplified using insulin specific primers and High Fidelity pfu (Figure 1(b)). PCR amplified proinsulin cDNA was cloned in pPICZ-*α*, under the control of strong and inducible AOX1 promoter. Moreover, proinsulin gene was cloned in frame with the native* S. cerevisiaeα*-factor signal sequence at N-terminus and polyhistidine tag at C-terminus to facilitate the efficient secretion and purification of recombinant protein as shown in Figure 1(a). Ligated mixture was transformed into competent* E. coli* cells. Several Zeocin resistant transformants were obtained on LB-Zeocin plate. Few clones were screened for the presence of proinsulin cDNA in pPICZ vector. Four clones were found to be positive as shown in Figure 2(a). These +ve clones were further confirmed by PCR using insulin specific primers (Figure 2(b)) and DNA sequencing.

### 3.2. Transformation of* Pichia pastoris*

**Figure 3:**
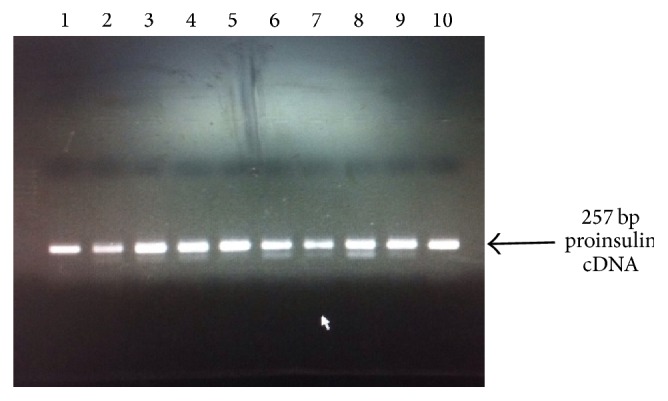
Agarose gel electrophoresis for checking the integration of proinsulin cDNA into* Pichia* genome. Transformation of pPICZ-C-insulin into* Pichia pastoris SuperMan*_*5*_ strain was done by electroporation and confirmation for integration of insulin cDNA in* Pichia* genome was done by PCR. Lanes 1 to 10: recombinant* Pichia* clones harboring pPICZ-*α*-proinsulin plasmid.

*Pichia pastoris SuperMan*_*5*_ strain was selected for expression of insulin protein. In this GlycoSwitch strain, glycan processing enzyme (OCH1) has been mutated to prevent glycan elongation and moreover, it overexpresses a heterologous mannosidase to cleave extra glycans to generate homogenous Man5 structure. This particular strain of* Pichia* synthesizes recombinant protein with more human-like glycosylation and hence it is more suitable for production of therapeutic proteins. Linearized form of pPICZ-*α*-insulin plasmid DNA was transformed into* Pichia pastoris SuperMan*_*5*_ strain by electroporation. Since* Pichia* expression vectors do not contain yeast origin of replication, linearization of plasmid facilitates efficient recombination and integration of DNA in* Pichia* genome. For selecting putative multicopy recombinants, transformants were further cultured in presence of very high concentration of Zeocin, that is, 1000 and 2000 *μ*g/mL Zeocin. Integration of insulin cDNA in* Pichia* genome was further confirmed by PCR as shown in Figure 3.

### 3.3. Expression and Purification of Insulin Protein

**Figure 4:**
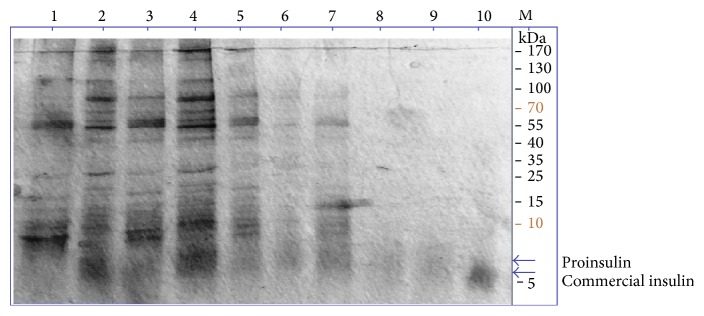
SDS-PAGE gel showing expression of recombinant human proinsulin in* Pichia pastoris*. Standard insulin (lane 10) and expressed human proinsulin at various time intervals (lanes 1–9), in* Pichia pastoris*, were run in 15% SDS-PAGE. About 10 *μ*L of transformed* P. pastoris* culture supernatants was loaded into lanes 1–9. As the image revealed, the expressed proinsulin had higher molecular weight (~8 KDa) in comparison with the standard one (lane 10), as it contains the C-peptide. The ~8 KDa band intensity seems increasing with the time intervals 24 h to 96 h as shown from lanes 9 to 1.

**Figure 5:**
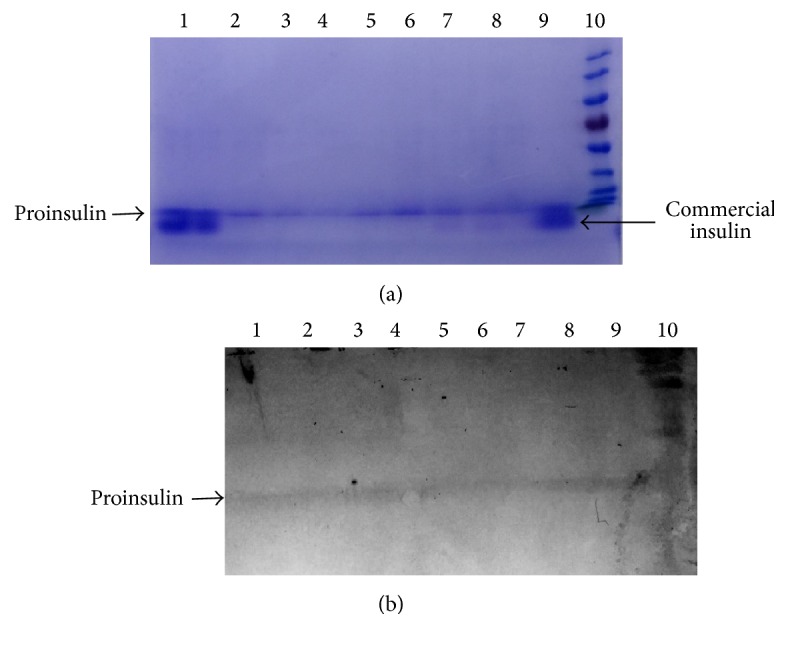
Western blot analysis to confirm the secreted expression of proinsulin in* Pichia pastoris*. (a) SDS-PAGE gel showing purified proinsulin protein. (b) Western blot analysis using insulin specific monoclonal antibodies.

Expression of insulin protein in* Pichia* clones was analyzed by ELISA and Western blot analysis using insulin specific monoclonal antibody. As insulin was fused with the yeast *α*–mating factor signal sequence at the N-terminus, recombinant insulin was detected in sufficient amount in culture supernatant (Figure 4). Expression of insulin was detected in* Pichia* clones by ELISA as well as by SDS-PAGE analysis as seen in Table 1 and Figures 4 and 5. As the Western blot image revealed, the expressed insulin had higher molecular weight (~8 KDa) in comparison with the standard one, due to the presence of C-chain in the recombinant insulin. The ^S^MUTs clone was observed to be superior over ^+^MUT* P. pastoris* clone for expression. The expressed and secreted insulin protein concentration as estimated with Bradford methods was 5 mg/L after 144 hrs of induction (Table 1).

### 3.4. Evaluation of the Activity of Recombinant Human Insulin in Diabetic Mice

**Figure 6:**
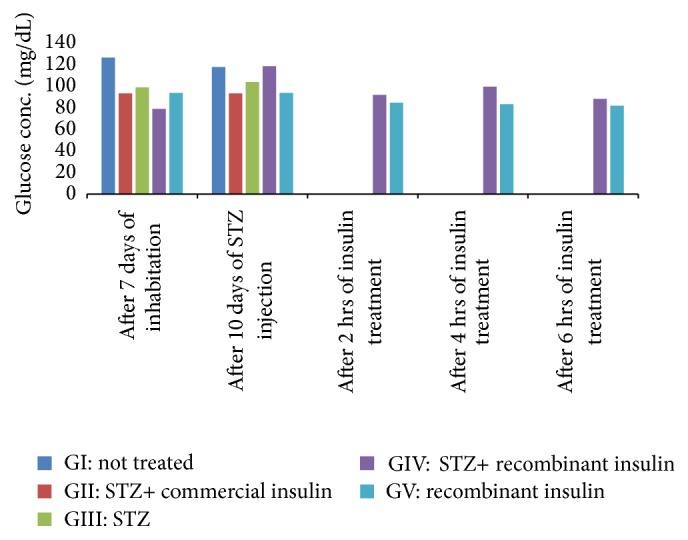
Evaluation of the efficacy of recombinant human insulin in diabetic mice. G: group; STZ: streptozotocin.

Biological activity of recombinant human insulin produced in* Pichia pastoris* was checked in streptozotocin induced diabetic mice. Our data indicated the efficacy of recombinant insulin in lowering the glucose level in diabetic mice as shown in Figure 6 (Group IV and Group V animals). Since recombinant proteins expressed in* Pichia* are properly folded, human insulin with intact C-peptide was found to be functionally active. However, in Group II, glucose levels could not be monitored, as all the animals died in this particular group.

Recombinant human insulin has been commercially produced using either* E. coli* or* Saccharomyces cerevisiae*. However, increasing cases of diabetes all over the world and development of new mode of insulin delivery such as oral or inhalation have led to increase in demand for recombinant insulin. There is an utmost need to improve the production capacity of current manufacturing technologies and also explore alternate expression host which can provide higher yield of recombinant insulin. Transgenic plants have potential to produce large amount of insulin at very low cost. Biologically active insulin has been shown to be expressed at very high levels in seeds or leaves of transgenic plants which provide additional advantage of long-term storage [17, 18]. In this study, we have explored the possibility of using methylotropic yeast* Pichia pastoris* for producing recombinant insulin. Several studies have reported the tremendous potential of* Pichia pastoris* to produce large amount of functionally active heterologous proteins [19–23]. Moreover, recombinant proteins are not hyperglycosylated in* Pichia*, which is seen as a major concern for the recombinant proteins being produced in* Saccharomyces cerevisiae*. Moreover, we have expressed the proinsulin along with the C-peptide, which is not present in the commercially available insulin. C-peptide had been previously thought to be biologically inactive, but few studies in last decade have shown the significance of C-peptide in treatment of diabetic complications such as nerve stimulation and renal functions. C-peptide had been shown to act independently of mature insulin by activating G-protein which causes influx of intracellular Ca^2+^ due to opening of Ca^2+^ channels and thus leads to activation of endothelial nitric oxide synthase, Na^+^,K^+^-ATPase and MAP kinase pathway [24]. Activation of Na^+^,K^+^-ATPase plays important role in restoration of renal function in diabetic patients [25] and activity of endothelial nitric oxide synthase and Na^+^,K^+^-ATPase is very essential for nerve function [26]. Boyhan and Daniell had reported the expression of human proinsulin containing all the three peptides, that is, A-, B-, and C-peptides, in tobacco leaves with approximate yield of 3 mg/g leaf. Delivery of proinsulin into mice resulted in reduction in blood glucose levels which was comparable to the commercial insulin [17]. Recent studies have suggested that inclusion of C-peptide would be more beneficial in long-term treatment of diabetic complications including impaired nerve and renal functions [24, 26].

## 4. Conclusion

In conclusion, we report here the expression and purification of biologically active proinsulin along with C-peptide, which offer some advantage over currently available insulins in managing long-term complications of diabetes. Moreover, we are in process of encapsulating the recombinant insulin in nanoparticles to develop oral insulin.

## Figures and Tables

**Table 1:** Expression of recombinant human insulin in *Pichia pastoris* as evaluated by ELISA.

Commercial insulin		Time (hrs)	Recombinant proinsulin			
Protein conc. (*µ*g/mL)	OD 495		Mut^+^ clone		Mut^S^ clone	
			OD 495	Protein conc. (*µ*g/mL)	OD 495	Protein conc. (*µ*g/mL)
5	0.695 ± 0.031	0	0.447 ± 0.015	—	0.473 ± 0.020	—
10	0.706 ± 0.015	24	0.523 ± 0.010	8	0.587 ± 0.120	11
15	0.776 ± 0.015	72	0.749 ± 0.023	20	0.0818 ± 0.021	26
20	0.798 ± 0.070	96	1.155 ± 0.130	31	1.205 ± 0.101	35
25	0.802 ± 0.110	144	0.870 ± 0.030	23	1.015 ± 0.060	29
30	0.870 ± 0.09					

Mut^+^: methanol utilization +ve clone.

Mut^S^: methanol utilization slow phenotype.
